# Transcriptome analysis reveals underlying immune response mechanism of fungal (*Penicillium oxalicum*) disease in *Gastrodia elata* Bl. *f. glauca* S. chow (Orchidaceae)

**DOI:** 10.1186/s12870-020-02653-4

**Published:** 2020-09-29

**Authors:** Yanhua Wang, Yugang Gao, Pu Zang, Yue Xu

**Affiliations:** College of Chinese Medicinal Materials, Jilin Agricultural University, Changchun, 130118 China

**Keywords:** *Gastrodia elata* Bl. *f. glauca* S. chow, Orchidaceae, Transcriptome, Fungal disease; immune response, Transcription factors, Changbai Mountain area

## Abstract

**Background:**

*Gastrodia elata* Bl. *f. glauca* S. Chow is a medicinal plant. *G. elata f. glauca* is unavoidably infected by pathogens in their growth process. In previous work, we have successfully isolated and identified *Penicillium oxalicum* from fungal diseased tubers of *G. elata f. glauca*. As a widespread epidemic, this fungal disease seriously affected the yield and quality of *G. elata f. glauca*. We speculate that the healthy *G. elata F. glauca* might carry resistance genes, which can resist against fungal disease. In this study, healthy and fungal diseased mature tubers of *G. elata f. glauca* from Changbai Mountain area were used as experimental materials to help us find potential resistance genes against the fungal disease.

**Results:**

A total of 7540 differentially expressed Unigenes (DEGs) were identified (FDR < 0.01, log2FC > 2). The current study screened 10 potential resistance genes. They were attached to transcription factors (TFs) in plant hormone signal transduction pathway and plant pathogen interaction pathway, including WRKY22, GH3, TIFY/JAZ, ERF1, WRKY33, TGA. In addition, four of these genes were closely related to jasmonic acid signaling pathway.

**Conclusions:**

The immune response mechanism of fungal disease in *G. elata f. glauca* is a complex biological process, involving plant hormones such as ethylene, jasmonic acid, salicylic acid and disease-resistant transcription factors such as WRKY, TGA.

## Background

*Gastrodia elata* Bl. *f. glauca* S. Chow is a form of *Gastrodia elata* Bl. (Orchidaceae). *G. elata* Bl., called *tian ma* in Chinese, is a perennial monocotyledon. Its dry tuber is usually used as a precious traditional Chinese medicine Gastrodiae Rhizoma. The main active ingredients of Gastrodiae Rhizoma include gastrodin, p-hydroxybenzyl alcohol, parishin E, parishin B, parishin C and parishin [1]. It is recorded that Gastrodiae Rhizoma has the functions of resting wind and relieving spasmodic, calming liver and inhibiting yang, dispelling wind and relaxing channels and collaterals [1]. Modern pharmacological research has shown that Gastrodiae Rhizoma has the effects of neuroregulation [2, 3], neuroprotection [4–7], improving memory [8, 9] and so on. It has auxiliary therapeutic effect on Alzheimer’s disease (AD) [8] and Parkinson’s disease (PD) [4, 6, 10, 11] which are the common degenerative diseases nowadays.

Six *G. elata* varietas were described in *Flora of Yunnan*, and they are *G. elata* Bl. f. *pilifera* Tuyama, *G. elata* Bl. *f. viridis* Makino, *G. elata* Bl. *f. glauca* S. Chow, *G. elata* Bl. *f. alba* S. Chow, *G. elata* Bl. *f. elata* and *G. elata* Bl. f. *flavida* S. Chow. They were respectively called as *Mao tian ma*, *Lv tian ma*, *Wu tian ma*, *Song tian ma*, *Hong tian ma*, *Huang tian ma* in Chinese. Among them, *G. elata F. glauca* is one of the most popular in the market because of its good shape and high dry rate. In China, *G. elata* Bl. *f. glauca* is mainly distributed in northeastern Yunnan, western Guizhou, southern Sichuan and Changbai Mountain area. *G. elata* Bl. *f. glauca* is not only a traditional Chinese medicinal material in Changbai Mountain, but also one of the most vital special economic crops in Jilin Province. However, the genetic research of *G. elata* Bl. *f. glauca* in Changbai Mountain area is almost blank.

*G. elata* Bl. is an obligate fungal heterotrophic plant with highly degraded leaves and bracts. More than 80% of its life cycle exists underground in the form of tuber, depending almost entirely on fungi to provide nutrient [12]. It is closely related to at least two types of fungi: *Mycena* to promote seed germination and *Armillaria Mellea* to ensure reproductive growth. The growth and development of *G. elata* Bl.usually goes through seed, protocorm, juvenile tuber (also called *Mi ma* in Chinese), immature tuber (also called *Bai ma* in Chinese), mature tuber (also called *Jian ma* in Chinese), scape, flower, and fruit [12]. During the growth and development of *G. elata*, it is susceptible to infection by non-essential fungi such as *Penicillium* [13], *Ilyonectria robusta* [14] and *Trichoderma hamatum* [15]. The main natural diseases that occur on *G. elata* Bl. *f. glauca* are soft rot, black spot and mildew. In our previous studies, two fungal pathogens (*Penicillium oxalicum*, *Candida vartiovaarae*) were isolated and identified from diseased *G. elata* Bl. *f. glauca*. Fungal disease induced by *Penicillium oxalicum* had widespread prevalence in Changbai Mountain area [13]. Diseased *G. elata* Bl. tubers become moldy, soft and rotted [13]. Fungal disease incidence in *G. elata* Bl. *f. glauca* is 6% ~ 17%, giving rise to a 10% ~ 30% reduction in yield [16]. So far, there is no research report on disease resistance breeding of *G. elata* Bl. *f. glauca* by means of genomics tools. Therefore, it is imperative to carry out research on immune response mechanism of fungal disease in *G. elata* Bl. *f. glauca*.

Obviously, under the same condition of being infected, physiologically healthy *G. elata* Bl. *f. glauca* probably have potential disease resistance genes. We intended to screen candidate genes for disease resistance through differential expression analysis. In this study, a detailed comparison was made between healthy and fungal diseased *G. elata* Bl. *f. glauca* tubers by means of transcriptome sequencing and bioinformatics analysis. It may provide a new insight for the breeding of disease resistant varieties of *G. elata* Bl. *f. glauca*.

## Results

### Sequencing overview

7.89 × 10^10^ base (healthy group) and 6.45 × 10^10^ base (fungal diseased group) clean data were generated by sequencing platform. GC content ranged from 47.16 to 49.09%, and Q30 of each sample was above 92.92% (Additional file: Table S1). It was showed that sequencing fragments had high randomness and reliability (Additional file: Figure S1A). After transcript de novo assembly, 60,324 Unigenes in total were obtained, and the N50 was 2409 kb. Furthermore, 19,670 (32.61%) of them were over 1 kb in length (Additional file: Figure S1B). All these indicative data displayed high assembly integrity.

### Functional annotation and differential expression analysis

#### DEGs annotation and function classification

**Fig. 1:**
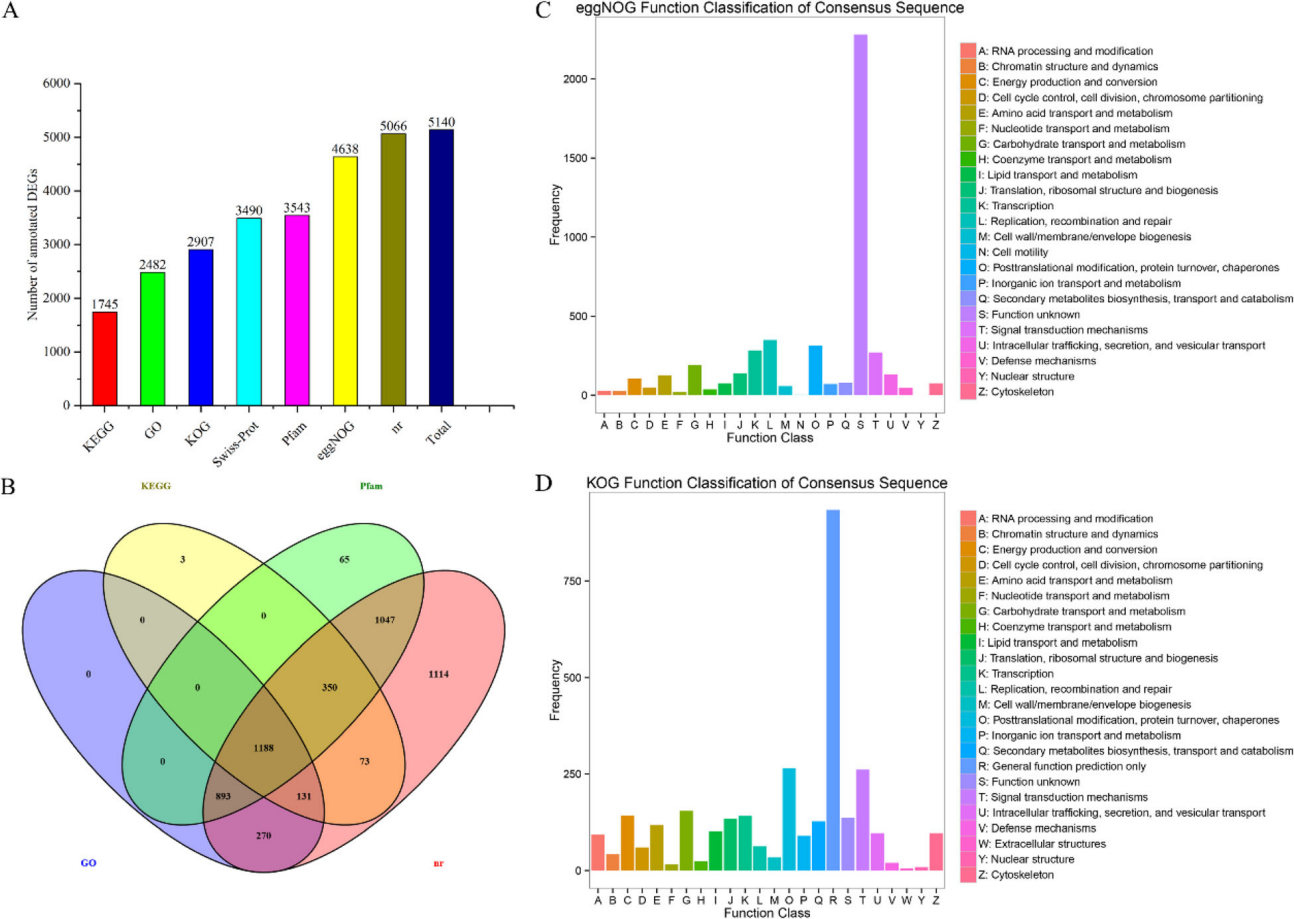
DEGs functional annotation information. **a** DEGs number annotated into KEGG, GO, KOG, Swiss-Prot, Pfam, eggNOG, nr and total number of annotated DEGs. **b** Venn diagram of DEGs number annotated into KEGG, GO, Pfam, nr. **c** Functional classification of DEGs annotated into eggNOG. **d** Functional classification of DEGs annotated into KOG. Capital letters A ~ Z represent different functional categories

The most DEGs annotated into nr (RefSeq non-redundant proteins), while the least annotated into KEGG (Fig. 1a). The venn diagram displayed the set of DEGs in four common databases which covered nearly all annotated DEGs (Fig. 1b). It was learned that DEGs between healthy and fungal diseased samples chiefly classified into “signal transduction mechanisms”, “carbohydrate transport and metabolism”, “defense mechanisms”, “energy production and conversion”, “general function prediction only”, “post-translation modification, protein turnover, chaperones”, “translation, ribosomal structure and biogenesis” (Fig. 1c, d).

### GO enrichment and KEGG enrichment analysis

**Fig. 2:**
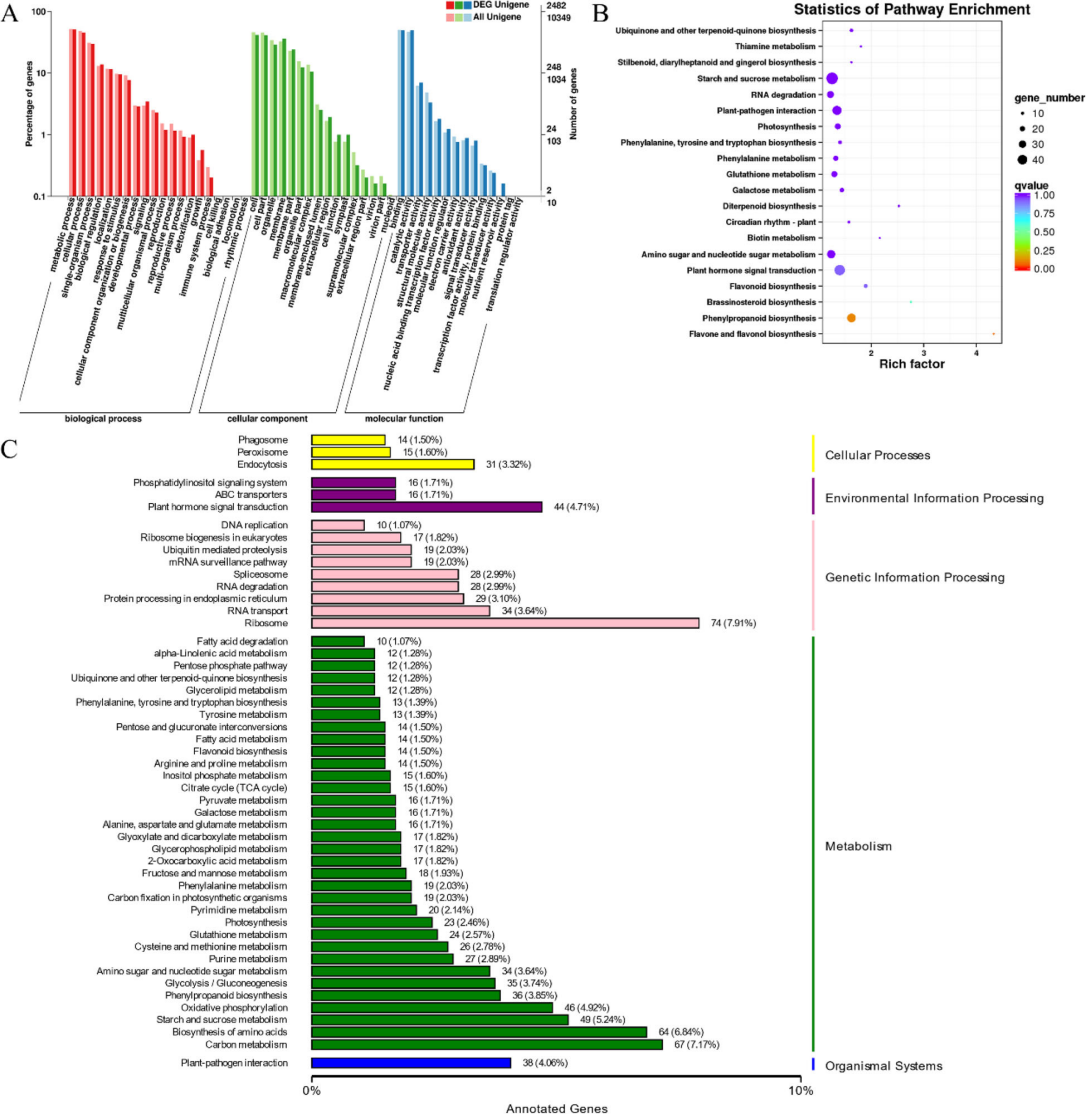
Unigenes function enrichment analysis. **a** Top 30 GO enriched function categories with the largest number of annotated Unigenes. **b** Statistics of KEGG pathway enrichment. Each circle represents a KEGG pathway. **c** Top 50 KEGG enriched function categories with the largest number of annotated Unigenes

2482 DEGs were enriched into 3958 GO terms. GO terms are usually classified into 3 categories: biological process (BP), cellular component (CC), molecular function (MF). Here, 2363 (59.70%) of these GO terms attached to BP, 509 (1.49%) belonged to CC, and 1086 (27.44%) were part of MF. 36 GO terms involved signal transduction, and 24 GO terms involved phytohormone. By Kolmogorov-Smirnov test, 421 GO terms were significantly enriched (*p* < 0.05). Part of them were showed in Additional file: Table S2 (*p* < 0.05) and top 30 were displayed as Fig. 2a.

122 pathways (Additional file: Table S3) were enriched and top 50 was showed as Fig. 2c. The enrichment degree was based on *p* value and enrichment factor (Fig. 2b). Nine pathways were significantly enriched (*p* < 0.05), and they attached to three pathway categories: metabolism, environmental information processing, organismal systems (Table 1).

**Table 1:** KEGG pathway enrichment analysis (*p* < 0.05)

Pathway category	Pathway description	Specific pathway	ko ID	DEG	All Unigene	*p*
Metabolism	Carbohydrate metabolism	Starch and sucrose metabolism	ko00500	49	169	0.041
	Metabolism of cofactors and vitamins	Ubiquinone and other terpenoid-quinone biosynthesis	ko00130	12	32	0.047
	Metabolism of terpenoids and polyketides	Brassinosteroid biosynthesis	ko00905	7	11	0.005
		Diterpenoid biosynthesis	ko00904	7	12	0.009
	Biosynthesis of other secondary metabolites	Flavone and flavonol biosynthesis	ko00944	5	5	0.001
		Phenylpropanoid biosynthesis	ko00940	36	96	0.001
		Flavonoid biosynthesis	ko00941	14	32	0.008
Environmental Information Processing	Signal transduction	Plant hormone signal transduction	ko04075	44	136	0.008
Organismal Systems	Environmental adaptation	Plant-pathogen interaction	ko04626	38	122	0.023

### Differential expression analysis

**Fig. 3:**
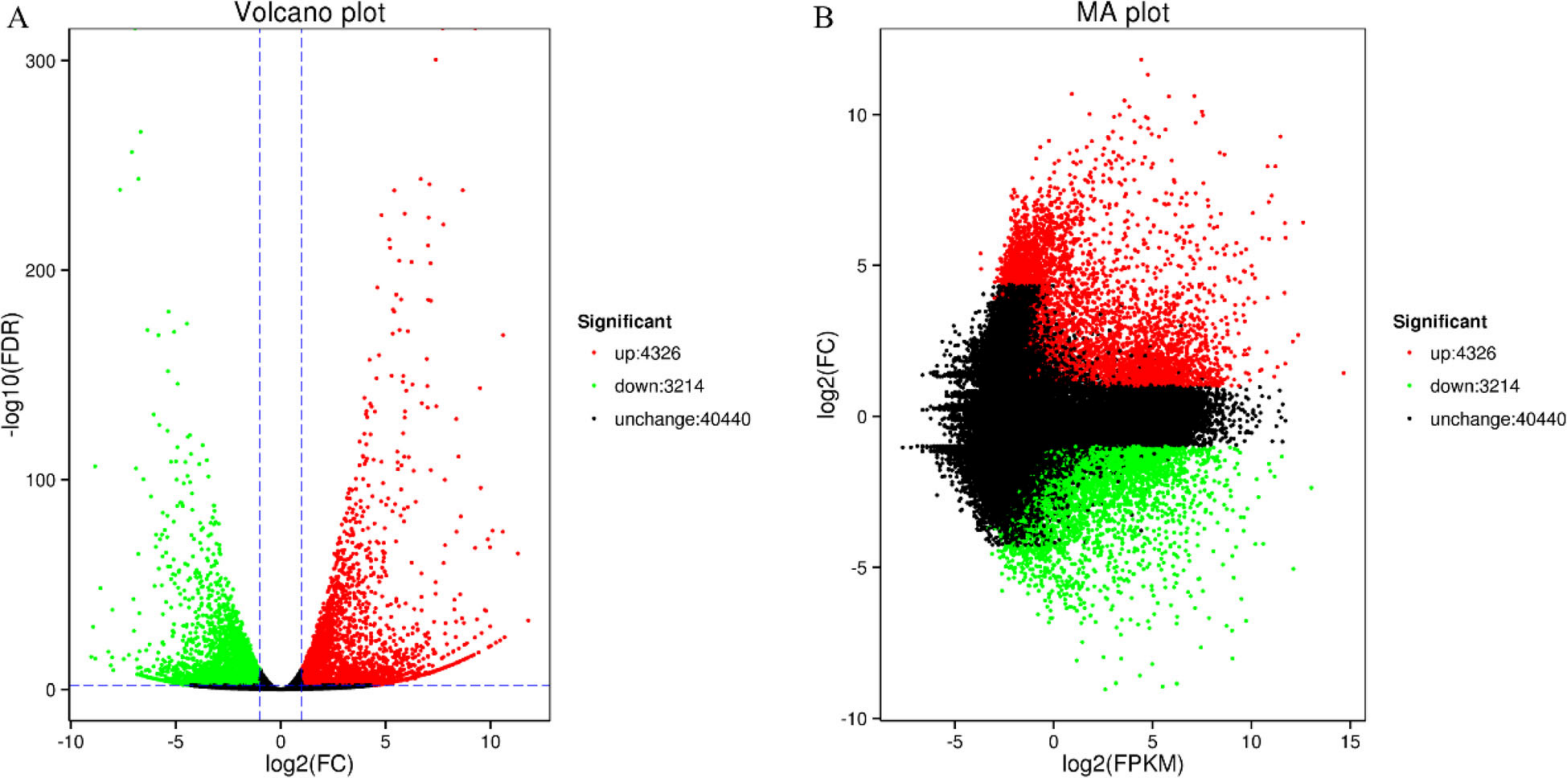
Differential expression analysis. Each dot represents a gene. Green represents down-regulation; red represents up-regulation; black represents non-differentially expression. **a** Volcano map of DEGs. X-axis represents the log2(FC) value. The greater the absolute value of log2(FC), the greater the difference of gene expression level between the two groups. Y-axis represents the negative log10(FDR) value. The larger the value, the more significant the difference, as well the more reliable the DEGs. **b** MA plot of DEGs. MA plot displays the normalized gene distribution. X-axis represents the log2(FPKM) value, and Y-axis represents the log2(FC) value

A total of 7540 DEGs were identified. 4326 of these DEGs were up-regulated in diseased group, and 3214 were down-regulated (Fig. 3a, b). In addition, 40,440 Unigenes did not demonstrate significantly differential expression. Overall, DEGs between healthy and diseased samples accounted for 15.71% of all Unigenes.

### Transcription factor prediction

**Fig. 4:**
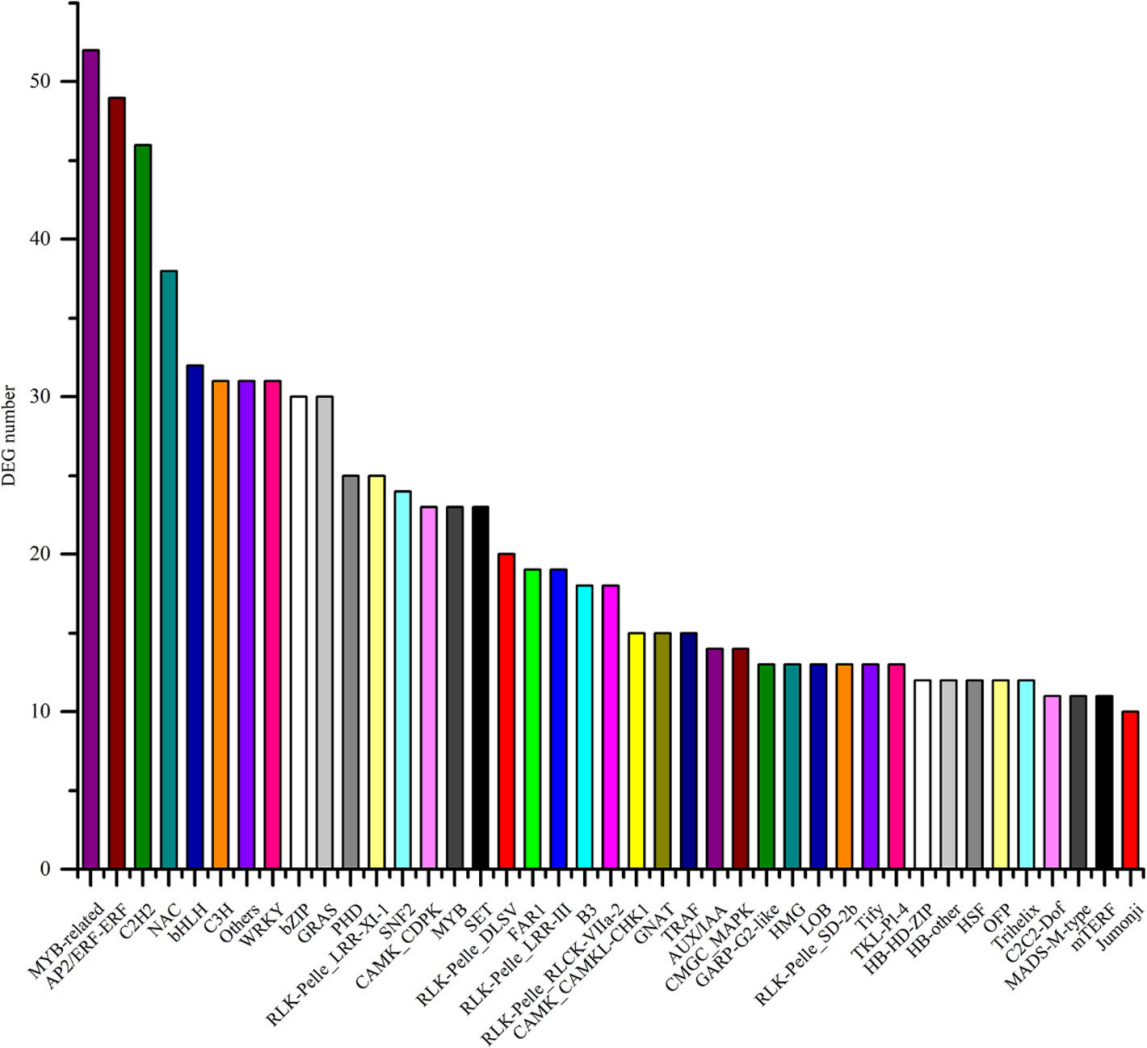
Transcription factor prediction. X-axis represents the names of transcription factor family, and Y-axis represents the number of DEGs

By the standard of FDR < 0.01 and FC > 2, 1295 DEGs were identified as transcription factors with transcription factor prediction tool (Fig. 4). Here, transcription factor family covers transcription factor (TF), transcription regulator (TR), protein kinases (PK). It could be clear to see that many DEGs were the members of transcription factor families MYB, ERF, C2H2, NAC, bHLH, C3H, WRKY, bZIP, GRAS, PHD, SNF2, SET.

### KEGG pathways analysis

**Fig. 5:**
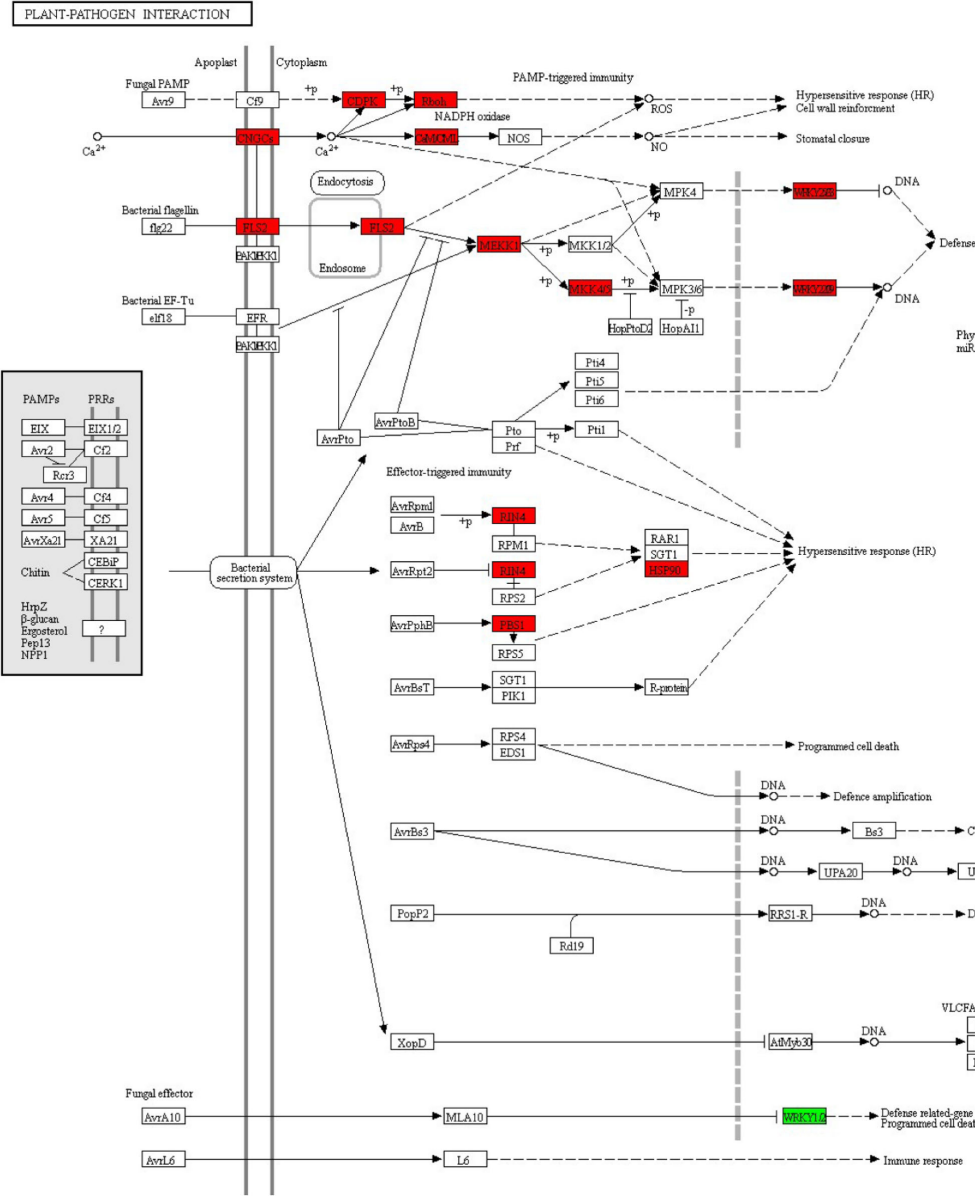
Plant-pathogen interaction map. Positive regulation is highlighted in red; negative regulation is highlighted in green

**Fig. 6:**
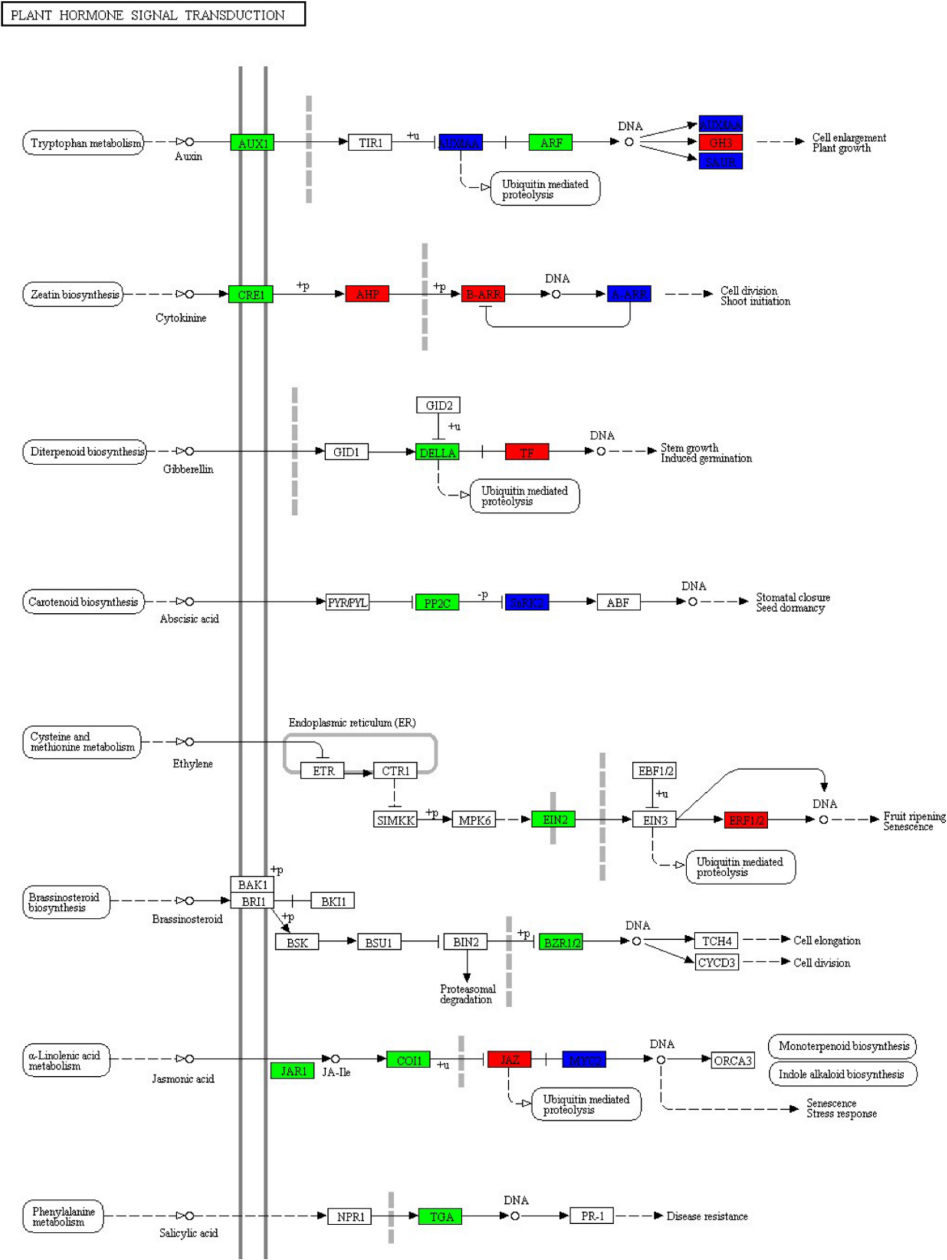
Plant hormone signal transduction map. Positive regulation is highlighted in red; negative regulation is highlighted in green; mixed regulation is highlighted in blue

**Fig. 7:**
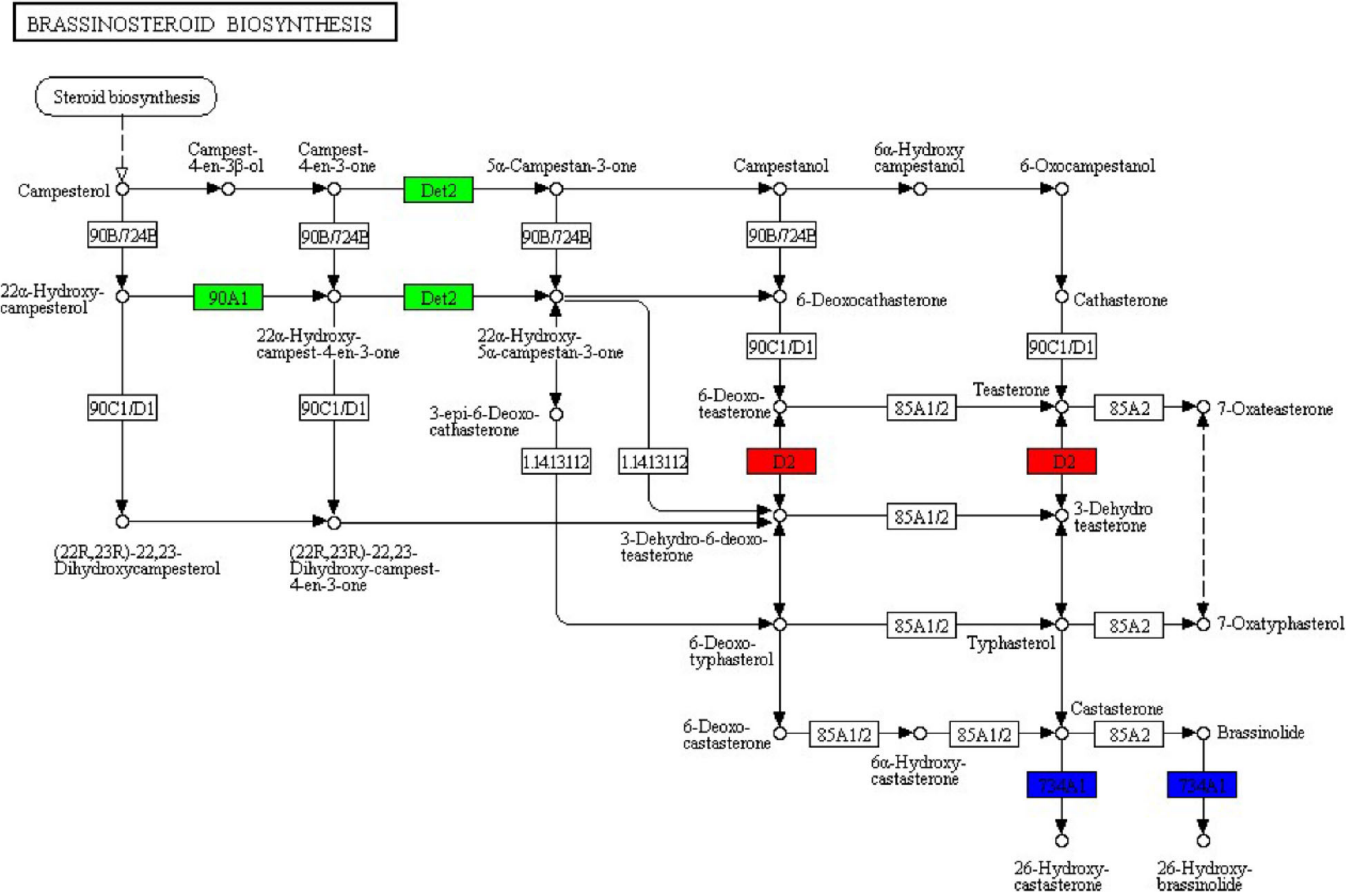
Brassinosteroid biosynthesis map. Positive regulation is highlighted in red; negative regulation is highlighted in green; mixed regulation is highlighted in blue

The current study paid close attention to pathways related to plant immune response. In plant-pathogen interaction map, only one node displayed negative regulation, and other 14 nodes revealed positive regulation (Fig. 5). In plant hormone signal transduction map, 6 nodes were up-regulated, 10 nodes were down-regulated, and 6 were mix-regulated (Fig. 6). In brassinosteroid biosynthesis map, 2 nodes showed positive regulation, 3 nodes displayed negative regulation, and 2 nodes covered both up-regulated genes and down-regulated genes (Fig. 7).

### Candidate genes responding to fungal disease in *G. elata* Bl. *f. glauca*

**Fig. 8:**
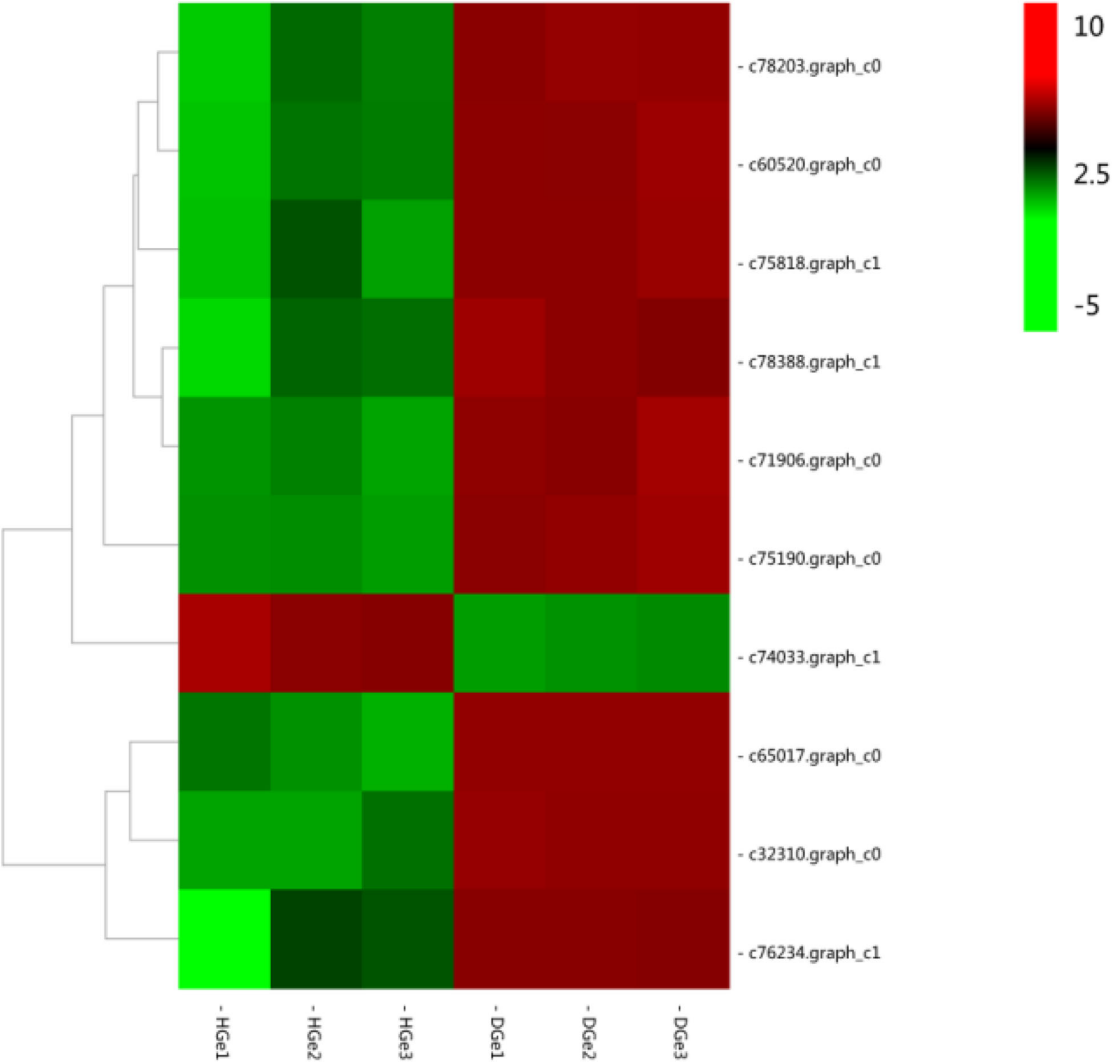
Cluster heatmap of immune response genes of fungal disease. Red indicates positive regulation and green indicates negative regulation. The gene expression levels are indicated by log2FPKM values and displayed in shades of color. The darker the color, the greater the log2FPKM value, and the higher the gene expression level

Comprehensively considering gene expression levels (FPKM> 10), significance of differential expression (FDR < 0.01, |log2FC| > 2) and literature related to plant immune response [17–21], 10 candidate genes responding to fungal disease in *G. elata* Bl. *f. glauca* were found (Fig. 8; Table 2).

**Table 2:** Information of disease resistance genes. ko04626: plant-pathogen interaction; ko04075: plant hormone signal transduction. K13425: WRKY22; K14487: GH3; K13464: JAZ; K13448: CML; K14516: ERF1; K13424: WRKY33; K14431: TGA

Gene ID	FDR	Log2FC	Regulated	pathway	KEGG entry	nr annotation
c65017.graph_c0	8.65E-170	10.60551	up	ko04626	K13425	probable WRKY transcription factor 27, partial [*Phalaenopsis equestris*]
c32310.graph_c0	7.67E-97	9.531941	up	ko04075	K14487	probable indole-3-acetic acid-amido synthetase GH3.1 [*Phalaenopsis equestris*]
c75818.graph_c1	5.48E-19	5.547811	up	ko04075	K13464	protein TIFY 10a-like [*Dendrobium catenatum*]
c76234.graph_c1	1.23E-15	5.462372	up	ko04075	K13464	protein TIFY 10c-like [*Dendrobium catenatum*]
c60520.graph_c0	1.11E-77	4.956140	up	ko04075	K13464	protein TIFY 10a-like [*Dendrobium catenatum*]
c75190.graph_c0	3.26E-110	4.430928	up	ko04626	K13448	probable calcium-binding protein CML18 [*Phalaenopsis equestris*]
c78203.graph_c0	7.49E-63	4.276427	up	ko04075	K14516	ethylene-responsive transcription factor 1B-like [*Dendrobium catenatum*]
c78388.graph_c1	1.21E-46	3.499401	up	ko04075	K13464	protein TIFY 10a-like [*Dendrobium catenatum*]
c71906.graph_c0	6.55E-51	2.652510	up	ko04626	K13424	WRKY transcription factor WRKY24-like isoform X1 [*Dendrobium catenatum*]
c74033.graph_c1	8.03E-27	−2.15672	down	ko04075	K14431	transcription factor TGA1-like [*Dendrobium catenatum*]

### Real-time quantitative polymerase chain reaction (qRT-PCR) analysis

Seven genes showed higher expression in the fungal diseased group (*p*<0.05), and one displayed negative expression (*p*<0.05). Only the gene labeled as c32310 revealed no significant difference in relative expression level between the two groups (*p*>0.05). In addition, there was no quantitative result for one gene, which may be due to unreasonable primer design.

## Discussion

### Pathways related to plant immune response

So far, it has been proved that plant immune response is relative to plant-pathogen interaction, plant hormone signal transduction, and pathways about certain secondary metabolite biosynthesis or metabolism [22–26]. Consistently, we got similar results in this study (Table 1).

In plant-pathogen interaction pathway, all except WRKY1/2 were up-regulated. They were CDPK (calcium-dependent protein kinase), Rboh (respiratory burst oxidase homolog), CNGC (cyclic nucleotide gated channel), calcium-binding protein CML (calmodulin-like protein), LRR (leucine-rich repeat) receptor-like serine/threonine-protein kinase FLS2, MEKK1 (mitogen-activated protein kinase kinase kinase 1), MKK4/5 (mitogen-activated protein kinase kinase 4/5), WRKY transcription factor 33, WRKY transcription factor 22, RIN4 (RPM1-interacting protein 4), serine/threonine-protein kinase PBS 1, molecular chaperone HtpG. Biological processes these up-regulated genes principally involved were hypersensitive response (HR), cell wall reinforcement, defense-related gene induction, phytoalexin accumulation and miRNA production. Some of these genes were involved in PAMP-triggered immunity. Only WRKY transcription factor 2 displayed down-regulated expression, and it was connected with HR, defense-related gene induction and programmed cell death.

In plant hormone signal transduction, we learned that GH3 (auxin responsive glycoside hydrolase 3 gene family), AHP (histidine-containing phosphotransfer protein), ARR-B (two-component response regulator ARR-B family), PIF4 (phytochrome-interacting factor 4), ERF1 (ethylene-responsive transcription factor 1), JAZ (jasmonate ZIM domain-containing protein) were up-regulated. AUX1 (auxin influx carrier), ARF (auxin response factor), CRE1 (cytokinin receptor enzyme), DELLA protein, PP2C (protein phosphatase 2C), EIN2 (ethylene-insensitive protein 2), BZR1/2 (brassinosteroid resistant 1/2), JAR1 (jasmonic acid-amino synthetase), COI1 (coronatine-insensitive protein 1), transcription factor TGA showed down-regulated. As it described, transcription factor TGA is connected with disease resistance [27]. DEGs in this pathway involved many biological processes, such as cell enlargement, plant growth, cell division, shoot initiation, stem growth, stomatal closure, seed dormancy, fruit ripening, senescence, monoterpenoid biosynthesis, indole alkaloid biosynthesis, cell elongation, of course, disease resistance as well (Fig. 6). Above biological processes usually accompanied by phosphorylation (+p), dephosphorylation (−p), ubiquitination (+u). Phosphorylation and ubiquitination are common post-translational modification of proteins. They play an important role in pattern-triggered immunity (PTI), and simultaneously be necessary to receptor complex activation signals and cell homeostasis [28]. Phytohormone played a vital role in this pathway. They included jasmonic acid (JA), salicylic acid (SA), ethylene (ET), brassinosteroid (BR), auxin, cytokinine, gibberellin, abscisic acid.

In fact, plant hormones do play a vital role in the process of plant-pathogen interaction. The current study found a large number of DEGs annotated to signal transduction mechanisms by means of functional annotation. Furthermore, lots of DEGs were markedly enriched into plant hormone signal transduction pathway. Consistently, it has been reported that auxin [29, 30], cytokinins [31, 32], ethylene [30, 33–35], gibberellin [36], abscisic acid [30, 37, 38], brassinosteroids [35], salicylic acid [30, 33, 39], jasmonic acid [30, 33, 39–41], strigolactones [42] can actively participate in disease response. Among them, salicylic acid signal transduction and jasmonic acid/ethylene signal transduction are considered as the most common plant hormone signal transduction pathways in response to biological or abiotic stress. It could even be said that the plant resistance against pathogen is initially stimulated by gene expression regulated by transcription factors and ultimately be mediated by plant hormones. Therefore, if possible, it is necessary to study phytohormone metabolism of *G. elata* Bl. *f. glauca* in the following work.

Brassinosteroid is one of crucial phytohormone closely related to plant growth and stress response. In brassinosteroid biosynthesis pathway, CYP90D2 (steroid 3-oxidase) showed up-regulated expression; CYP90A1 (cytochrome P450 family 90 subfamily A polypeptide 1) displayed down-regulated expression; CYP734A1/BAS1 (PHYB activation tagged suppressor 1) was mix-regulated, with two genes up-regulated and one gene down-regulated (Fig. 7).

The current study also found numerous DEGs appear in the pathways of secondary metabolites biosynthesis. CYP75B1 and CYP75A showed significant differential expression in flavone and flavonol biosynthesis pathway. 4CL, CYP84A appeared in phenylpropanoid biosynthesis pathway. 4CL and CYP73A displayed positive regulation in ubiquinone and other terpenoid-quinone biosynthesis pathway. 4CL is a key enzyme in the synthesis of lignin and it can response to osmotic stress by regulating secondary cell wall development and stomatal [43]. This may be a part of fungal disease immune response mechanism in *G. elata* Bl. *f. glauca.*

In starch and sucrose metabolism pathway, DEGs involved in fructose and glucose synthesis were mainly positively regulated, and they were fructokinase (EC:2.7.1.4), beta-fructofuranosidase (EC:3.2.1.26), hexokinase (EC:2.7.1.1), phosphoglucomutase (EC:5.4.2.2) and UTP-glucose-1-phosphate uridylyltransferase (EC:2.7.7.9); while several DEGs involved in starch and glycogen synthesis mainly showed negative regulation, and they covered 1,4-alpha-glucan branching enzyme (EC:2.4.1.18), starch synthase (EC:2.4.1.21), 4-alpha-glucanotransferase (EC:2.4.1.25) and so on.

In summary, fungal disease immune response is a complex process involving multiple biological processes. It covers more than one gene and one gene does not work in single pathway. That is to say, one gene may perform more than one function simultaneously. These significantly enriched pathways might well reveal the underlying immune response mechanism of fungal disease in *G. elata* Bl. *f. glauca*.

### Defense-related transcription factors

It has been proved that many a transcription factor could directly or indirectly regulate plants immune response [26, 44–62]. Here, the current study got the similar result (Fig. 4). Exceptionally, according to transcription factor prediction, some C3H genes were differentially expressed in two groups. However, present reports about C3H are mainly related to cold resistance, rather than disease resistance [63, 64].

### Resistance genes (R genes)

**Fig. 9:**
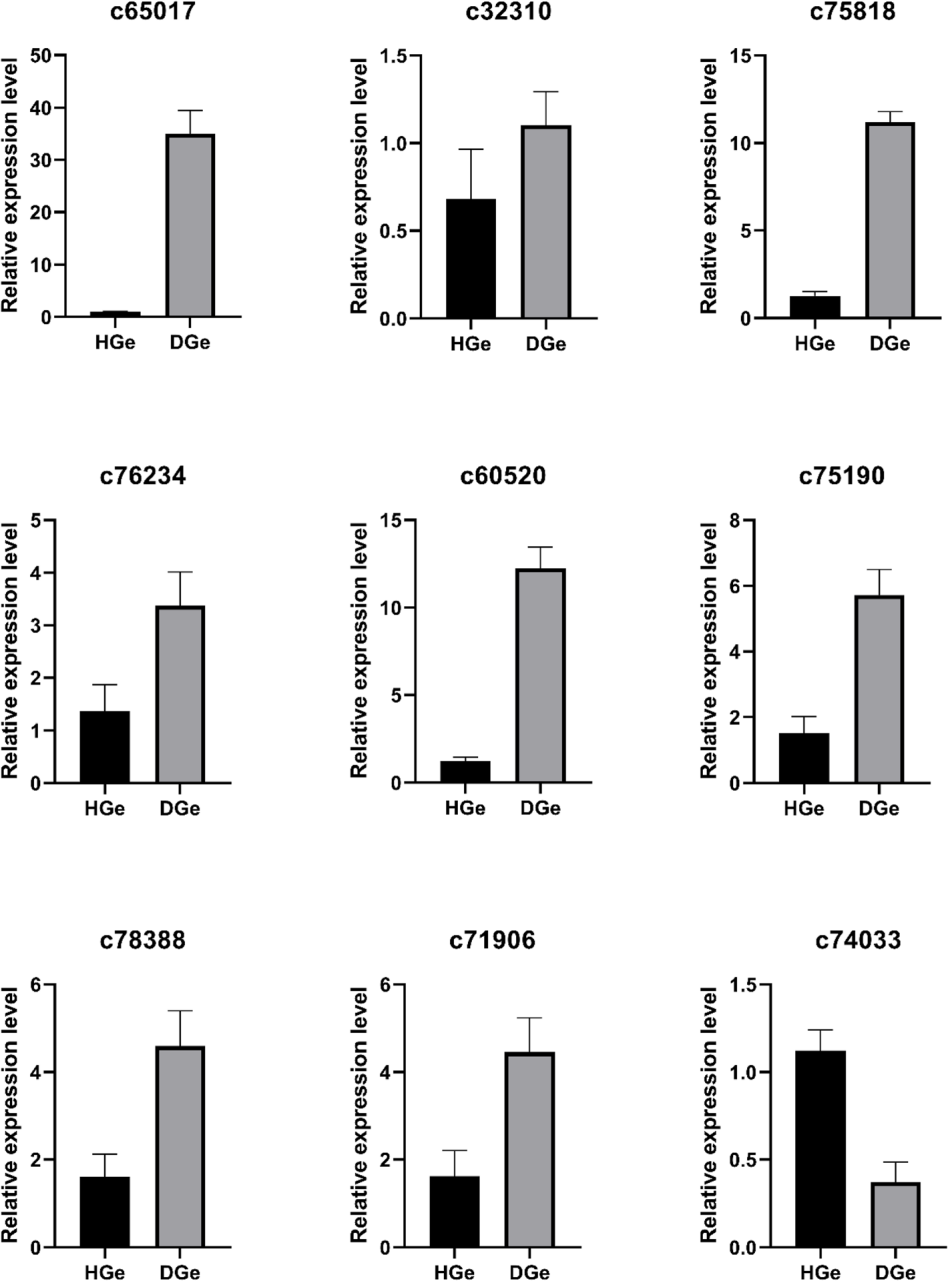
Relative expression levels of nine potential immune response genes by qRT-PCR assays. The relative expression levels are displayed with the 2^-ΔΔCt^ values. All genes but c32310 show significant differential expression between HGe and DGe groups (*p* < 0.05)

Resistance genes (R genes) were classified into nine types based on intracellular and extracellular pathogen recognition mechanisms [65]. Here, the current study discovered potential R genes in *G. elata* Bl. *f. glauca* were probably the member of transcription factor families like WRKY, GH3, TIFY/JAZ, CML, ERF, TGA. Coincidently, it has been reported that above transcription factors do be widely involved in various defense responses [26, 66–78]. It is reported that GH3 and CML can also regulate fruit development [79, 80]. To verify the accuracy of transcriptome sequencing, qRT-PCR test was performed, and the results were basically consistent with transcriptome sequencing (Fig. 9). However, it still needs further study on how these genes perform their functions in respond to fungal disease in *G. elata* Bl. *f. glauca*.

### Potential immune response mechanism of fungal disease in *G. elata* Bl. *f. glauca*

Plant immune response mechanisms mainly include PAMP-triggered immunity (PTI), effector-triggered immunity (ETI) and systemic acquired resistance (SAR). ETI is usually accompanied by the occurrence of hypersensitivity reaction (HR), giving rise to programmed cell death (PCD). Moreover, ETI can induce SAR. As is known to all, PTI and SAR are non-specific immunity, while ETI is specific immunity [81]. From the current study, the immune response mechanism of fungal disease in *G. elata* Bl. *f. glauca* involves all above three kinds of mechanisms in the whole process of infection.

**Fig. 10:**
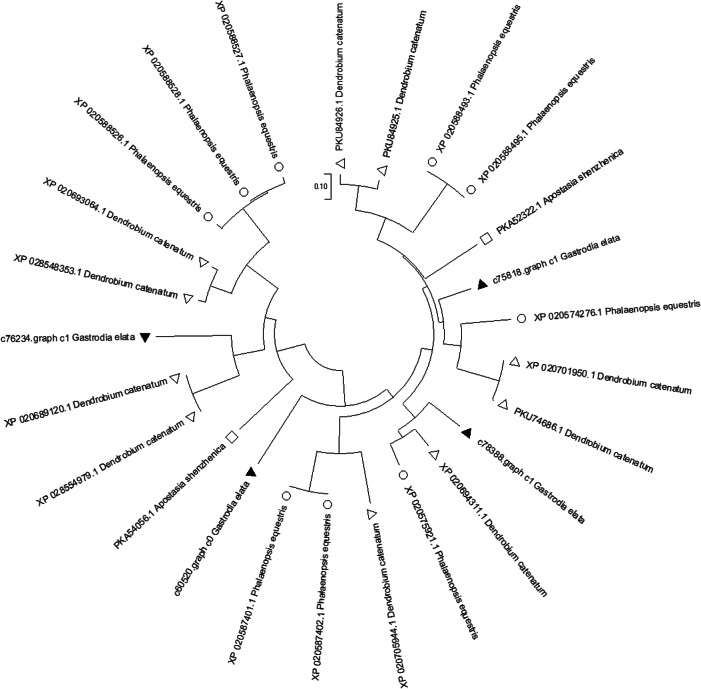
Phylogenetic tree of TIFY10 in Orchidaceae plants. Branch length represents the credibility of homology. The shorter the branch, the higher the credibility of homology. Different species are displayed with different symbols. ▲(solid triangle): *G. elata* Bl. *f. glauca*; △(hollow triangle): *Dendrobium catenatum*; ○(circle): *Phalaenopsis equestris*; □(square): *Apostasia shenzhenica*

In this study, many genes related to stress response and disease resistance demonstrated high expression and significant difference. They were members of certain transcription factor families, like WRKY, GH3, JAZ, CML, ERF, TGA. Furthermore, these genes were closely connected with derivatives of jasmonic acid, salicylic acid, brassinosteroid, ethylene and auxin. By BLAST (https://blast.ncbi.nlm.nih.gov/Blast.cgi), it is revealed that amino acid sequences of four JAZ genes in *G. elata* family were highly similar to certain gene sequences in *Dendrobium catenatum*, *Phalaenopsis equestris*, *Apostasia shenzhenica* (Fig. 10). They were all belong to TIFY10 family.

## Conclusions

In conclusion, immune response mechanism of fungal disease in *G. elata* Bl. *f. glauca* is quite complicated. JA/ET signal transduction and SA signal transduction show positive regulation in this progress. Firstly, the expression of JAZ and ERF1 positively induces ubiquitin mediated proteolysis. Secondly, the expression of TGA indirectly triggered disease resistance in physiologically healthy group, rather than in diseased group. Thirdly, brassinosteroid biosynthesis also makes contributions to fungal disease response. CYP90A1 and CYP90D2 display down-regulation and up-regulation, respectively. Last but not least, auxin signaling pathway involves in fungal disease response actively. However, JA/ET signaling pathway is undoubtedly the most highlighted. As the candidate genes response to fungal disease in *G. elata* Bl. *f. glauca*, their specific functions still need to be further verified. Of course, more insight into the molecular mechanisms of fungal disease response also requires to be revealed. If possible, we intend to perform transgenic functional verification of these candidate genes.

## Methods

### Plant material and growth conditions

Healthy tubers (HGe, Accession ID: SAMN14380862) and fungal diseased tubers (DGe, Accession ID: SAMN14380861) were used in this experiment [13]. These experimental materials were all from the Changbai Mountain area. They were identified as mature tubers of *G. elata* Bl. *f. glauca* S. Chow in Jilin Agricultural University.

Fresh tubers were collected in October, 2018 from a planting base (126°44′20″E, 42°24′30″N) attached to JINGZHEN TIANMA Co., Ltd. It is located in Jingyu County, Baishan City, Jilin Province, PR China. The manager of this company gave permission for sampling. Jingyu County is located in the western foot of Changbai Mountain and the upper reaches of Songhua River, PR China, with average altitude 775 m, annual average temperature 2.5 °C, effective accumulated temperature 2224 °C, annual average rainfall 767.3 mm, frost-free period 110 d or so. According to data from China Meteorological Administration (http://data.cma.cn/data/weatherBk.html), the monthly mean temperature range from − 17 °C to 21 °C, the monthly relative humidity ranged from 58 to 83%, and the monthly rainfall ranged from 7.8 to 207.4% (Additional file: Figure. S2). The vegetative growth of *G. elata* Bl. *f. glauca* is usually from April of the first year to October of the following year or even longer. However, it usually takes only from April to June for it to complete the reproductive growth process. And it takes about 1.5 ~ 3 years to mature for harvest the tuber [82]. The soil type of local area where they grow is dark brown soils on hillside.

### RNA extraction

Fresh *G. elata* Bl. *f. glauca* tubers used for RNA extraction were washed with sterile water, and after surface disinfection, 100 mg or so healthy tissue was cut near the infected tissue from diseased tubers. Tissues were taken from the same part of healthy tubers to keep uniformity between the two samples, each of which has three biological replications. The total RNA was extracted from each tissue using RNAprep Pure Plant Total RNA Extraction Kit (Polysaccharides & Polyphenolics-rich) (centrifugal column type, catalog No. DP441) and referring to the manual on its official website (https://www.tiangen.com/). RNA was quantified in an Implen NanoPhotometer N50 ultra-micro ultraviolet spectrophotometer (Thermo Scientific). The purity and integrity of RNA was determined in an Agilent 2100 Bioanalyzer. Finally, qualified total RNA was obtained, and the quality indicators were shown in Additional file: Table S4.

### cDNA library construction and sequencing

Follow steps were required to build the library: purification and fragmentation of mRNA, synthesis and purification of double-stranded cDNA, the end repair or dA tail addition, junction ligation and USER (uracil-specific excision reagent) enzyme digestion, ligated products purification and fragments size classification, library amplification, magnetic bead purification or sorting of amplification products, library quality control [83]. cDNA library was checked for quality and quantity using Agilent 2100 Bioanalyzer. All RNA sequences of 150 bp between 5′-terminal and 3′-terminal was sequenced through Illumina Noveseq high-flux sequencing platform [83]. Paired-end sequencing data was generated for each sample with 2 × 150 bps read lengths.

### Reads mapping and transcript de novo assembly

The resulting reads called raw data were stored in fastq format. The raw data of each sequencing sample included two fastq files containing reads determined at both ends of all cDNA fragments. The quality of raw reads was assessed using the fastqc program (http://www.bioinformatics.babraham.ac.uk/projects/fastqc/). Data filtering on raw data to remove low quality reads and reads containing connector or poly-N, we obtained high quality clean data.

Using Trinity software (https://github.com/trinityrnaseq/trinityrnaseq/wiki) with default parameters, the sequence assembly of clean data is carried out in combined assembly [84]. In this way, the sequencing depth can be increased indirectly, and transcripts with low expression abundance in *G. elata* Bl. *f. glauca* RNA samples can be assembled more completely. Clean data of each sample was aligned with assembled transcript or Unigene library to obtain mapped reads that matched transcript or Unigene library.

### Gene expression and annotation

A Unigene supported by a minimum of three mapped high-quality reads was considered as expressed. This was done for the sake of reducing the false positive caused by independent statistical hypothesis test to a large number of gene expression values. FC (fold change) means the ratio of gene expression levels between healthy and diseased groups. Positive values show upregulation and negative values show down regulation of genes in diseased group. In addition, FPKM (reads per kilobase of exon model per million mapped reads) value is also a factor to be considered for DEGs identification. When gene expression abundance is small, that is to say, be with low signal values, it may not be detected in subsequent validation.

In organisms, different genes perform different biological functions, similar genes have similar functions. In order to predict the function of unknown genes and obtain their functional annotation information, all Unigenes were annotated into databases such as GO (Gene Ontology) [85], KEGG (Kyoto Encyclopedia of Genes and Genomes) [86], COG (clusters of orthologous groups) [87], KOG (clusters of euKaryotic Orthologous Groups) [88], eggNOG (Evolutionary Genealogy of Genes: Non-supervised Orthologous Groups) [89].

### qRT-PCR

Using LightCycler® 480 II real-time PCR system (Roche, Switzerland) and 2X SG Fast qPCR Master Mix (B639271, BBI, Canada), the expression levels of 9 genes in healthy and diseased *G. elata* Bl. *f. glauca* were relatively quantified. The primer sequences (Additional file: Table S5) were designed using Primer 5.0 and synthesized by Sangon Biotech (Shanghai) Co., Ltd. (https://www.sangon.com). A two-step procedure (hold 95 °C 3 min; 45 × (duration 95 °C 5 s, anneal/extend 60 °C 30 s)) was used for qRT-PCR assays. Each sample contained three biological replicates. The relative expression values were calculated with the 2^-ΔΔCt^ method and normalized by the internal reference gene 18S rRNA (https://www.ncbi.nlm.nih.gov/nuccore/PVEL01000548[90, 91].

### Statistical analysis

The data in this study was shown as the mean values of three biological duplication. Pearson correlation coefficient is used when discussing samples correlation [92] (Additional file: Table S6). DEGs were evaluated with the DESeq2 package (http://www.bioconductor.org/packages/release/bioc/html/DESeq.html). Benjamini-Hochberg method was used to correct the significant *p* obtained from the original hypothesis test. The gene expression abundance was described by FPKM value. In addition, the differential expression and enrichment analysis were conducted using Fisher′s exact test to obtain an adjusted *p* with an FDR correction.

## Supplementary information

**Additional file 1: Table S1.** Sequencing and reads mapping.

**Additional file 2: Table S2.** GO terms enrichment of DEGs. KS: Kolmogorov-Smirnov test (*p*<0.01).

**Additional file 3: Table S3.** Enriched KEGG pathways.

**Additional file 4: Table S4.** Concentration, purity and integrity of total RNA.

**Additional file 5: Table S5.** Primer pair sequences for qRT-PCR.

**Additional file 6: Table S6.** Correlation statistics between biological replicate samples. It is revealed with Pearson′s correlation coefficient *r*. The closer *r*^2^ is to 1, the stronger the correlation between two groups.

**Additional file 7: Figure S1.** (a) Base distribution and reads average rate of raw data. (b) Transcripts and Unigenes length distribution after de novo assembly.

**Additional file 8: Figure S2.** Monthly values accumulated from 1981 to 2010 in Jingyu County, Baishan City, Jilin Province, PR China.

## Data Availability

The sequence data generated during the current study are available in the NCBI SRA repository via accession numbers SAMN14380862 and SAMN14380861 (https://www.ncbi.nlm.nih.gov/bioproject/PRJNA612737). All data analyzed during this study are included in this published article and its additional files.

## Abbreviations

DEG: Differentially expressed Unigene
TF: Transcription factor
AD: Alzheimer’s disease
PD: Parkinson’s disease
BP: Biological process
CC: Cellular component
MF: Molecular function
KS: Kolmogorov-Smirnov test
nr: RefSeq non-redundant proteins
GO: Gene Ontology
KEGG: Kyoto Encyclopedia of Genes and Genomes
COG: Clusters of orthologous groups
KOG: Clusters of euKaryotic Orthologous Groups
eggNOG: Evolutionary Genealogy of Genes: Non-supervised Orthologous Groups
TR: Transcription regulator
PK: Protein kinases
CDPK: Calcium-dependent protein kinase
Rboh: Respiratory burst oxidase homolog
CNGC: Cyclic nucleotide gated channel
CML: Calmodulin-like protein
LRR: Leucine-rich repeat
MEKK1: Mitogen-activated protein kinase kinase kinase 1
MKK4/5: Mitogen-activated protein kinase kinase 4/5
RIN4: RPM1-interacting protein 4
HR: Hypersensitive response
GH3: Glycoside hydrolase 3
AHP: Histidine-containing phosphotransfer protein
ARR-B: Two-component response regulator ARR-B family
PIF4: Phytochrome-interacting factor 4
ERF1: Ethylene-responsive transcription factor 1
JAZ: Jasmonate ZIM domain-containing protein
AUX1: Auxin influx carrier
ARF: Auxin response factor
CRE: Cytokinin receptor enzyme
PP2C: Protein phosphatase 2C
EIN2: Ethylene-insensitive protein 2
BZR1/2: Brassinosteroid resistant 1/2
JAR1: Jasmonic acid-amino synthetase
COI1: Coronatine-insensitive protein 1
+p: Phosphorylation
-p: Dephosphorylation
+u: Ubiquitination
PTI: Pattern-triggered immunity
JA: Jasmonic acid
SA: Salicylic acid
ET: Ethylene
BR: Brassinosteroid
ETI: Effector-triggered immunity
SAR: Systemic acquired resistance
R genes: Resistance genes
HGe: Healthy tubers
DGe: Diseased tubers
FDR: False discovery rate
FC: Fold change
