# Supplementary material for: Transcriptome analysis reveals underlying immune response mechanism of fungal (Penicillium oxalicum) disease in Gastrodia elata Bl. f. glauca S. chow (Orchidaceae)
Source: BMC Plant Biol. 2020 Sep 29;20:445. doi: 10.1186/s12870-020-02653-4 (PMC7525978; doi:10.1186/s12870-020-02653-4)
Supplement: Supplementary file 1 — Additional file 1: Table S1. Sequencing and reads mapping. [file 12870_2020_2653_MOESM1_ESM.docx]

**Table S1** Sequencing and reads mapping.

| Sample ID | Read number | Base number | GC (%) | Q30 (%) | Mapped reads | Mapped ratio |
| --- | --- | --- | --- | --- | --- | --- |
| HGe_1 | 25470344 | 7592321670 | 47.16 | 93.45 | 20094596 | 78.89% |
| HGe_2 | 28113884 | 8374746564 | 47.18 | 93.36 | 22090546 | 78.58% |
| HGe_3 | 25917830 | 7705332808 | 47.71 | 93.82 | 20663785 | 79.73% |
| DGe_1 | 22292030 | 6635386288 | 48.44 | 92.92 | 17137138 | 76.88% |
| DGe_2 | 20862769 | 6230732256 | 48.54 | 93.39 | 16234156 | 77.81% |
| DGe_3 | 21654775 | 6470230374 | 49.09 | 93.17 | 17038768 | 78.68% |
