# Supplementary material for: Transcriptome analysis reveals underlying immune response mechanism of fungal (Penicillium oxalicum) disease in Gastrodia elata Bl. f. glauca S. chow (Orchidaceae)
Source: BMC Plant Biol. 2020 Sep 29;20:445. doi: 10.1186/s12870-020-02653-4 (PMC7525978; doi:10.1186/s12870-020-02653-4)
Supplement: Supplementary file 2 — Additional file 2: Table S2. GO terms enrichment of DEGs. KS: Kolmogorov-Smirnov test (p<0.01). [file 12870_2020_2653_MOESM2_ESM.docx]

**Table S2** GO terms enrichment of DEGs. BP: biological process; CC: cellular component; MF: molecular function; KS: Kolmogorov-Smirnov test (*p*<0.01).

| Process | GO ID | Term description | Annotated Unigene | DEGs | *p* |
| --- | --- | --- | --- | --- | --- |
| BP | GO:0055114 | oxidation-reduction process | 929 | 199 | 4.60E-6 |
| BP | GO:0006468 | protein phosphorylation | 352 | 111 | 0.00011 |
| BP | GO:0006097 | glyoxylate cycle | 12 | 2 | 0.00084 |
| BP | GO:0016310 | phosphorylation | 628 | 204 | 0.00148 |
| BP | GO:0015992 | proton transport | 146 | 37 | 0.00227 |
| BP | GO:0009698 | phenylpropanoid metabolic process | 26 | 10 | 0.00228 |
| BP | GO:0006355 | regulation of transcription, DNA-templated | 416 | 107 | 0.00239 |
| BP | GO:0042775 | mitochondrial ATP synthesis coupled electron transport | 32 | 10 | 0.00262 |
| BP | GO:0006032 | chitin catabolic process | 18 | 5 | 0.00268 |
| BP | GO:0044550 | secondary metabolite biosynthetic process | 45 | 17 | 0.00335 |
| BP | GO:0015979 | photosynthesis | 114 | 35 | 0.00379 |
| BP | GO:0072350 | tricarboxylic acid metabolic process | 13 | 3 | 0.00394 |
| BP | GO:0009809 | lignin biosynthetic process | 7 | 6 | 0.00405 |
| BP | GO:0055085 | transmembrane transport | 535 | 142 | 0.00448 |
| BP | GO:0009084 | glutamine family amino acid biosynthetic process | 42 | 7 | 0.00466 |
| BP | GO:0010143 | cutin biosynthetic process | 4 | 4 | 0.00520 |
| BP | GO:0051248 | negative regulation of protein metabolic process | 23 | 2 | 0.00628 |
| BP | GO:0051503 | adenine nucleotide transport | 5 | 4 | 0.00739 |
| BP | GO:0015868 | purine ribonucleotide transport | 5 | 4 | 0.00739 |
| BP | GO:0030029 | actin filament-based process | 33 | 6 | 0.00755 |
| BP | GO:0006096 | glycolytic process | 77 | 17 | 0.00759 |
| BP | GO:0010268 | brassinosteroid homeostasis | 4 | 4 | 0.00767 |
| BP | GO:0051338 | regulation of transferase activity | 35 | 7 | 0.00779 |
| BP | GO:0032012 | regulation of ARF protein signal transduction | 5 | 4 | 0.00842 |
| BP | GO:0031505 | fungal-type cell wall organization | 16 | 5 | 0.00858 |
| BP | GO:0001932 | regulation of protein phosphorylation | 33 | 5 | 0.00884 |
| BP | GO:0043934 | sporulation | 16 | 3 | 0.00926 |
| BP | GO:0065009 | regulation of molecular function | 158 | 37 | 0.00975 |
| CC | GO:0016020 | membrane | 3364 | 900 | 1.00E-5 |
| CC | GO:0005576 | extracellular region | 173 | 48 | 6.60E-5 |
| CC | GO:0005829 | cytosol | 759 | 140 | 0.00036 |
| CC | GO:0016021 | integral component of membrane | 2134 | 548 | 0.00121 |
| CC | GO:0005618 | cell wall | 120 | 42 | 0.00271 |
| CC | GO:0032153 | cell division site | 28 | 3 | 0.00274 |
| CC | GO:0044445 | cytosolic part | 280 | 48 | 0.00275 |
| CC | GO:0009521 | photosystem | 78 | 26 | 0.00296 |
| CC | GO:0000329 | fungal-type vacuole membrane | 12 | 0 | 0.00297 |
| CC | GO:0005758 | mitochondrial intermembrane space | 13 | 5 | 0.00408 |
| CC | GO:0070469 | respiratory chain | 69 | 20 | 0.00637 |
| CC | GO:0045121 | membrane raft | 5 | 3 | 0.00862 |
| MF | GO:0020037 | heme binding | 137 | 44 | 3.40E-6 |
| MF | GO:0004674 | protein serine/threonine kinase activity | 270 | 97 | 5.40E-5 |
| MF | GO:0005506 | iron ion binding | 124 | 29 | 0.00023 |
| MF | GO:0046912 | transferase activity, transferring acyl groups, acyl groups converted into alkyl on transfer | 25 | 4 | 0.00038 |
| MF | GO:0019829 | cation-transporting ATPase activity | 83 | 26 | 0.00060 |
| MF | GO:0003824 | catalytic activity | 4846 | 1228 | 0.00070 |
| MF | GO:0004497 | monooxygenase activity | 113 | 34 | 0.00081 |
| MF | GO:0048037 | cofactor binding | 315 | 72 | 0.00105 |
| MF | GO:0016491 | oxidoreductase activity | 964 | 218 | 0.00158 |
| MF | GO:0016705 | oxidoreductase activity, acting on paired donors, with incorporation or reduction of molecular oxygen | 120 | 36 | 0.00173 |
| MF | GO:0003993 | acid phosphatase activity | 16 | 7 | 0.00178 |
| MF | GO:0004568 | chitinase activity | 18 | 5 | 0.00244 |
| MF | GO:0008061 | chitin binding | 18 | 3 | 0.00244 |
| MF | GO:0015662 | ATPase activity, coupled to transmembrane movement of ions, phosphorylative mechanism | 30 | 15 | 0.00289 |
| MF | GO:0005524 | ATP binding | 986 | 270 | 0.00297 |
| MF | GO:0004672 | protein kinase activity | 418 | 149 | 0.00304 |
| MF | GO:0000287 | magnesium ion binding | 83 | 28 | 0.00326 |
| MF | GO:0016740 | transferase activity | 1743 | 494 | 0.00337 |
| MF | GO:0004197 | cysteine-type endopeptidase activity | 29 | 8 | 0.00351 |
| MF | GO:0030234 | enzyme regulator activity | 96 | 24 | 0.00466 |
| MF | GO:0016878 | acid-thiol ligase activity | 12 | 6 | 0.00541 |
| MF | GO:0016405 | CoA-ligase activity | 12 | 6 | 0.00541 |
| MF | GO:0005086 | ARF guanyl-nucleotide exchange factor activity | 7 | 5 | 0.00622 |
| MF | GO:0016653 | oxidoreductase activity, acting on NAD(P)H, heme protein as acceptor | 6 | 2 | 0.00661 |
| MF | GO:0016717 | oxidoreductase activity, acting on paired donors, with oxidation of a pair of donors resulting in the reduction of molecular oxygen to two molecules of water | 14 | 6 | 0.00691 |
| MF | GO:0030599 | pectinesterase activity | 14 | 8 | 0.00789 |
| MF | GO:0003700 | transcription factor activity, sequence-specific DNA binding | 173 | 45 | 0.00885 |
