# Supplementary material for: Transcriptome analysis reveals underlying immune response mechanism of fungal (Penicillium oxalicum) disease in Gastrodia elata Bl. f. glauca S. chow (Orchidaceae)
Source: BMC Plant Biol. 2020 Sep 29;20:445. doi: 10.1186/s12870-020-02653-4 (PMC7525978; doi:10.1186/s12870-020-02653-4)
Supplement: Supplementary file 3 — Additional file 3: Table S3. Enriched KEGG pathways. [file 12870_2020_2653_MOESM3_ESM.docx]

**Table S3** Enriched KEGG pathways.

| Pathway name | ko ID | DEG | Unigene | *p* |
| --- | --- | --- | --- | --- |
| Flavone and flavonol biosynthesis | ko00944 | 5 | 5 | 0.00065 |
| Phenylpropanoid biosynthesis | ko00940 | 36 | 96 | 0.000918 |
| Brassinosteroid biosynthesis | ko00905 | 7 | 11 | 0.004651 |
| Flavonoid biosynthesis | ko00941 | 14 | 32 | 0.007535 |
| Plant hormone signal transduction | ko04075 | 44 | 136 | 0.007557 |
| Diterpenoid biosynthesis | ko00904 | 7 | 12 | 0.008957 |
| Plant-pathogen interaction | ko04626 | 38 | 122 | 0.023419 |
| Starch and sucrose metabolism | ko00500 | 49 | 169 | 0.040922 |
| Ubiquinone and other terpenoid-quinone biosynthesis | ko00130 | 12 | 32 | 0.046799 |
| Photosynthesis | ko00195 | 23 | 73 | 0.060238 |
| Galactose metabolism | ko00052 | 16 | 48 | 0.067959 |
| Biotin metabolism | ko00780 | 4 | 8 | 0.088823 |
| Glutathione metabolism | ko00480 | 24 | 80 | 0.091335 |
| Amino sugar and nucleotide sugar metabolism | ko00520 | 34 | 119 | 0.093655 |
| Phenylalanine metabolism | ko00360 | 19 | 62 | 0.104099 |
| Phenylalanine, tyrosine and tryptophan biosynthesis | ko00400 | 13 | 40 | 0.111484 |
| Circadian rhythm - plant | ko04712 | 8 | 22 | 0.112568 |
| Thiamine metabolism | ko00730 | 5 | 12 | 0.120397 |
| RNA degradation | ko03018 | 28 | 99 | 0.131909 |
| Stilbenoid, diarylheptanoid and gingerol biosynthesis | ko00945 | 6 | 16 | 0.14203 |
| Tyrosine metabolism | ko00350 | 13 | 42 | 0.151087 |
| Tropane, piperidine and pyridine alkaloid biosynthesis | ko00960 | 7 | 20 | 0.15754 |
| Linoleic acid metabolism | ko00591 | 5 | 13 | 0.160136 |
| Cyanoamino acid metabolism | ko00460 | 8 | 25 | 0.201269 |
| Oxidative phosphorylation | ko00190 | 46 | 178 | 0.209866 |
| Homologous recombination | ko03440 | 10 | 33 | 0.21333 |
| Glycerophospholipid metabolism | ko00564 | 17 | 61 | 0.226007 |
| Glycosphingolipid biosynthesis - globo series | ko00603 | 2 | 4 | 0.229812 |
| Anthocyanin biosynthesis | ko00942 | 1 | 1 | 0.230864 |
| Photosynthesis - antenna proteins | ko00196 | 9 | 30 | 0.240503 |
| Ribosome biogenesis in eukaryotes | ko03008 | 17 | 62 | 0.248698 |
| Glycolysis / Gluconeogenesis | ko00010 | 35 | 136 | 0.257124 |
| Carotenoid biosynthesis | ko00906 | 7 | 23 | 0.268089 |
| Taurine and hypotaurine metabolism | ko00430 | 3 | 8 | 0.274385 |
| Glycosaminoglycan degradation | ko00531 | 3 | 8 | 0.274385 |
| DNA replication | ko03030 | 10 | 35 | 0.275803 |
| Zeatin biosynthesis | ko00908 | 4 | 12 | 0.292759 |
| Fructose and mannose metabolism | ko00051 | 18 | 68 | 0.294356 |
| Inositol phosphate metabolism | ko00562 | 15 | 56 | 0.300733 |
| Fatty acid elongation | ko00062 | 6 | 20 | 0.306574 |
| Biosynthesis of unsaturated fatty acids | ko01040 | 8 | 28 | 0.309721 |
| Sulfur metabolism | ko00920 | 8 | 28 | 0.309721 |
| Lipoic acid metabolism | ko00785 | 2 | 5 | 0.326857 |
| Glycerolipid metabolism | ko00561 | 12 | 45 | 0.336822 |
| alpha-Linolenic acid metabolism | ko00592 | 12 | 45 | 0.336822 |
| Other types of O-glycan biosynthesis | ko00514 | 3 | 9 | 0.345772 |
| Isoquinoline alkaloid biosynthesis | ko00950 | 6 | 21 | 0.352446 |
| Phosphatidylinositol signaling system | ko04070 | 16 | 64 | 0.404661 |
| Cysteine and methionine metabolism | ko00270 | 26 | 107 | 0.419011 |
| Fatty acid biosynthesis | ko00061 | 8 | 31 | 0.427021 |
| Arginine and proline metabolism | ko00330 | 14 | 57 | 0.446223 |
| Pantothenate and CoA biosynthesis | ko00770 | 5 | 19 | 0.455656 |
| Nucleotide excision repair | ko03420 | 10 | 41 | 0.481625 |
| ABC transporters | ko02010 | 16 | 67 | 0.486053 |
| Fatty acid degradation | ko00071 | 10 | 42 | 0.5157 |
| Regulation of autophagy | ko04140 | 7 | 29 | 0.51842 |
| Nitrogen metabolism | ko00910 | 7 | 29 | 0.51842 |
| Folate biosynthesis | ko00790 | 4 | 16 | 0.523891 |
| Arachidonic acid metabolism | ko00590 | 4 | 16 | 0.523891 |
| Alanine, aspartate and glutamate metabolism | ko00250 | 16 | 69 | 0.539331 |
| 2-Oxocarboxylic acid metabolism | ko01210 | 17 | 74 | 0.555144 |
| Biosynthesis of amino acids | ko01230 | 64 | 281 | 0.575533 |
| Cutin, suberine and wax biosynthesis | ko00073 | 4 | 17 | 0.576441 |
| Pentose and glucuronate interconversions | ko00040 | 14 | 62 | 0.587663 |
| Sphingolipid metabolism | ko00600 | 4 | 18 | 0.625522 |
| Glycosphingolipid biosynthesis - ganglio series | ko00604 | 1 | 4 | 0.650201 |
| Vancomycin resistance | ko01502 | 1 | 4 | 0.650201 |
| Sesquiterpenoid and triterpenoid biosynthesis | ko00909 | 2 | 9 | 0.65162 |
| Pyrimidine metabolism | ko00240 | 20 | 92 | 0.661799 |
| Fatty acid metabolism | ko01212 | 14 | 65 | 0.664728 |
| Endocytosis | ko04144 | 31 | 142 | 0.67333 |
| Mismatch repair | ko03430 | 6 | 29 | 0.690695 |
| C5-Branched dibasic acid metabolism | ko00660 | 2 | 10 | 0.710428 |
| Vitamin B6 metabolism | ko00750 | 2 | 10 | 0.710428 |
| Non-homologous end-joining | ko03450 | 2 | 10 | 0.710428 |
| Valine, leucine and isoleucine biosynthesis | ko00290 | 4 | 20 | 0.712187 |
| Purine metabolism | ko00230 | 27 | 127 | 0.722907 |
| Porphyrin and chlorophyll metabolism | ko00860 | 6 | 30 | 0.723813 |
| Synthesis and degradation of ketone bodies | ko00072 | 1 | 5 | 0.731037 |
| Pentose phosphate pathway | ko00030 | 12 | 59 | 0.740198 |
| Steroid biosynthesis | ko00100 | 5 | 26 | 0.751173 |
| Protein export | ko03060 | 6 | 31 | 0.754371 |
| Basal transcription factors | ko03022 | 6 | 32 | 0.782383 |
| One carbon pool by folate | ko00670 | 3 | 17 | 0.788827 |
| Nicotinate and nicotinamide metabolism | ko00760 | 3 | 17 | 0.788827 |
| Lysine biosynthesis | ko00300 | 2 | 12 | 0.803189 |
| Other glycan degradation | ko00511 | 5 | 28 | 0.809198 |
| RNA transport | ko03013 | 34 | 167 | 0.828063 |
| Base excision repair | ko03410 | 6 | 34 | 0.831031 |
| Monobactam biosynthesis | ko00261 | 2 | 13 | 0.838847 |
| Valine, leucine and isoleucine degradation | ko00280 | 8 | 45 | 0.848728 |
| Tryptophan metabolism | ko00380 | 6 | 36 | 0.870559 |
| Ether lipid metabolism | ko00565 | 3 | 20 | 0.874011 |
| RNA polymerase | ko03020 | 5 | 31 | 0.875426 |
| Riboflavin metabolism | ko00740 | 1 | 8 | 0.877786 |
| Carbon metabolism | ko01200 | 67 | 325 | 0.880043 |
| Terpenoid backbone biosynthesis | ko00900 | 8 | 47 | 0.881237 |
| beta-Alanine metabolism | ko00410 | 7 | 42 | 0.883597 |
| SNARE interactions in vesicular transport | ko04130 | 3 | 21 | 0.894745 |
| Arginine biosynthesis | ko00220 | 8 | 49 | 0.907747 |
| Selenocompound metabolism | ko00450 | 3 | 22 | 0.912359 |
| Carbon fixation in photosynthetic organisms | ko00710 | 19 | 105 | 0.914096 |
| Peroxisome | ko04146 | 15 | 86 | 0.920685 |
| Aminoacyl-tRNA biosynthesis | ko00970 | 7 | 46 | 0.93247 |
| Glycine, serine and threonine metabolism | ko00260 | 10 | 63 | 0.941043 |
| Citrate cycle (TCA cycle) | ko00020 | 15 | 89 | 0.942302 |
| AGE-RAGE signaling pathway in diabetic complications | ko04933 | 1 | 11 | 0.944504 |
| Lysine degradation | ko00310 | 4 | 31 | 0.949806 |
| Propanoate metabolism | ko00640 | 5 | 37 | 0.951191 |
| Ubiquitin mediated proteolysis | ko04120 | 19 | 113 | 0.961061 |
| Butanoate metabolism | ko00650 | 2 | 20 | 0.963567 |
| Phagosome | ko04145 | 14 | 89 | 0.968031 |
| Pyruvate metabolism | ko00620 | 16 | 100 | 0.970059 |
| Ascorbate and aldarate metabolism | ko00053 | 5 | 40 | 0.970497 |
| mRNA surveillance pathway | ko03015 | 19 | 118 | 0.977322 |
| Spliceosome | ko03040 | 28 | 165 | 0.979974 |
| Glyoxylate and dicarboxylate metabolism | ko00630 | 17 | 110 | 0.982878 |
| Proteasome | ko03050 | 6 | 51 | 0.987455 |
| Histidine metabolism | ko00340 | 1 | 17 | 0.98858 |
| N-Glycan biosynthesis | ko00510 | 4 | 43 | 0.994801 |
| Protein processing in endoplasmic reticulum | ko04141 | 29 | 196 | 0.998824 |
| Ribosome | ko03010 | 74 | 429 | 0.999264 |
