# Supplementary material for: Transcriptome analysis reveals underlying immune response mechanism of fungal (Penicillium oxalicum) disease in Gastrodia elata Bl. f. glauca S. chow (Orchidaceae)
Source: BMC Plant Biol. 2020 Sep 29;20:445. doi: 10.1186/s12870-020-02653-4 (PMC7525978; doi:10.1186/s12870-020-02653-4)
Supplement: Supplementary file 4 — Additional file 4: Table S4. Concentration, purity and integrity of total RNA. [file 12870_2020_2653_MOESM4_ESM.docx]

**Table S4** Concentration, purity and integrity of total RNA.

| Sample ID | RIN | 28S/18S | OD260/280 | OD260/230 | Sample state |
| --- | --- | --- | --- | --- | --- |
| HGe_1 | 10.00 | 1.74 | 2.12 | 2.36 | normal |
| HGe_2 | 10.00 | 1.60 | 2.13 | 2.34 | normal |
| HGe_3 | 10.00 | 1.64 | 2.13 | 2.34 | normal |
| DGe_1 | 10.00 | 1.68 | 2.12 | 2.12 | normal |
| DGe_2 | 10.00 | 1.49 | 2.13 | 2.17 | normal |
| DGe_3 | 10.00 | 1.54 | 2.12 | 2.19 | normal |
