# Supplementary material for: Transcriptome analysis reveals underlying immune response mechanism of fungal (Penicillium oxalicum) disease in Gastrodia elata Bl. f. glauca S. chow (Orchidaceae)
Source: BMC Plant Biol. 2020 Sep 29;20:445. doi: 10.1186/s12870-020-02653-4 (PMC7525978; doi:10.1186/s12870-020-02653-4)
Supplement: Supplementary file 5 — Additional file 5: Table S5. Primer pair sequences for qRT-PCR. [file 12870_2020_2653_MOESM5_ESM.docx]

**Table S5** Primer pair sequences for qRT-PCR.

| Genes ID | Forward primer | Reverse primer |
| --- | --- | --- |
| 18S | GTACAAAGGGCAGGGACGTA | CCAGGTCCAGACATAGTAAG |
| c32310 | GCCTCCTCCGAGTGCTATTT | TAGTCATGGCCGAGCTTAACA |
| c60520 | TCCTGGCAATGAAGTCGC | GAGCACTTTCCCGTCGTAGA |
| c65017 | TCAGCCAAGCAATCGCAC | TCTGCCCGTACTTTCTCCAA |
| c71906 | AGAACAAGTCAGAGGACGGGTAT | TGACCGTTGAAGTTCCTCTCTAC |
| c74033 | CAGAATAGGCAGATTTGTGAGC | CAGAGGATTTCCATAAACCAGAC |
| c75190 | ATGGTGACGGAAAGATAACAGC | GCGTCCTTGATATCGGCAT |
| c75818 | AGGGATTTTCATCTCATAACGC | CAAGAACCTTCCCTCCGTAGA |
| c76234 | TGATGGAAATGGCAAGCAG | GAAAGGGTTTTACCTGAAGCTC |
| c78388 | ATGTCCCAGCTGACGATATTCTAC | CGATGCCTTCCTTGCGATA |
