# Supplementary material for: Transcriptome analysis reveals underlying immune response mechanism of fungal (Penicillium oxalicum) disease in Gastrodia elata Bl. f. glauca S. chow (Orchidaceae)
Source: BMC Plant Biol. 2020 Sep 29;20:445. doi: 10.1186/s12870-020-02653-4 (PMC7525978; doi:10.1186/s12870-020-02653-4)
Supplement: Supplementary file 6 — Additional file 6: Table S6. Correlation statistics between biological replicate samples. It is revealed with Pearson′s correlation coefficient r. The closer r2 is to 1, the stronger the correlation between two groups. [file 12870_2020_2653_MOESM6_ESM.docx]

**Table S6** Correlation statistics between biological replicate samples. It was revealed with Pearson′s correlation coefficient *r*. The closer *r*^2^ is to 1, the stronger the correlation between the two samples.

| *r* | HGe_1 | HGe_2 | HGe_3 | DGe_1 | DGe_2 | DGe_3 |
| --- | --- | --- | --- | --- | --- | --- |
| HGe_1 | 1 | 0.9308 | 0.8161 | 0.3807 | 0.3904 | 0.3845 |
| HGe_2 | 0.9308 | 1 | 0.7643 | 0.3090 | 0.3184 | 0.3046 |
| HGe_3 | 0.8161 | 0.7643 | 1 | 0.2513 | 0.2565 | 0.2508 |
| DGe_1 | 0.3807 | 0.3090 | 0.2513 | 1 | 0.9989 | 0.9952 |
| DGe_2 | 0.3904 | 0.3184 | 0.2565 | 0.9989 | 1 | 0.9956 |
| DGe_3 | 0.3845 | 0.3046 | 0.2508 | 0.9952 | 0.9956 | 1 |
