# Supplementary material for: Transcriptome analysis reveals underlying immune response mechanism of fungal (Penicillium oxalicum) disease in Gastrodia elata Bl. f. glauca S. chow (Orchidaceae)
Source: BMC Plant Biol. 2020 Sep 29;20:445. doi: 10.1186/s12870-020-02653-4 (PMC7525978; doi:10.1186/s12870-020-02653-4)
Supplement: Supplementary file 7 — Additional file 7: Figure S1. (a) Base distribution and reads average rate of raw data. (b) Transcripts and Unigenes length distribution after de novo assembly. [file 12870_2020_2653_MOESM7_ESM.docx]

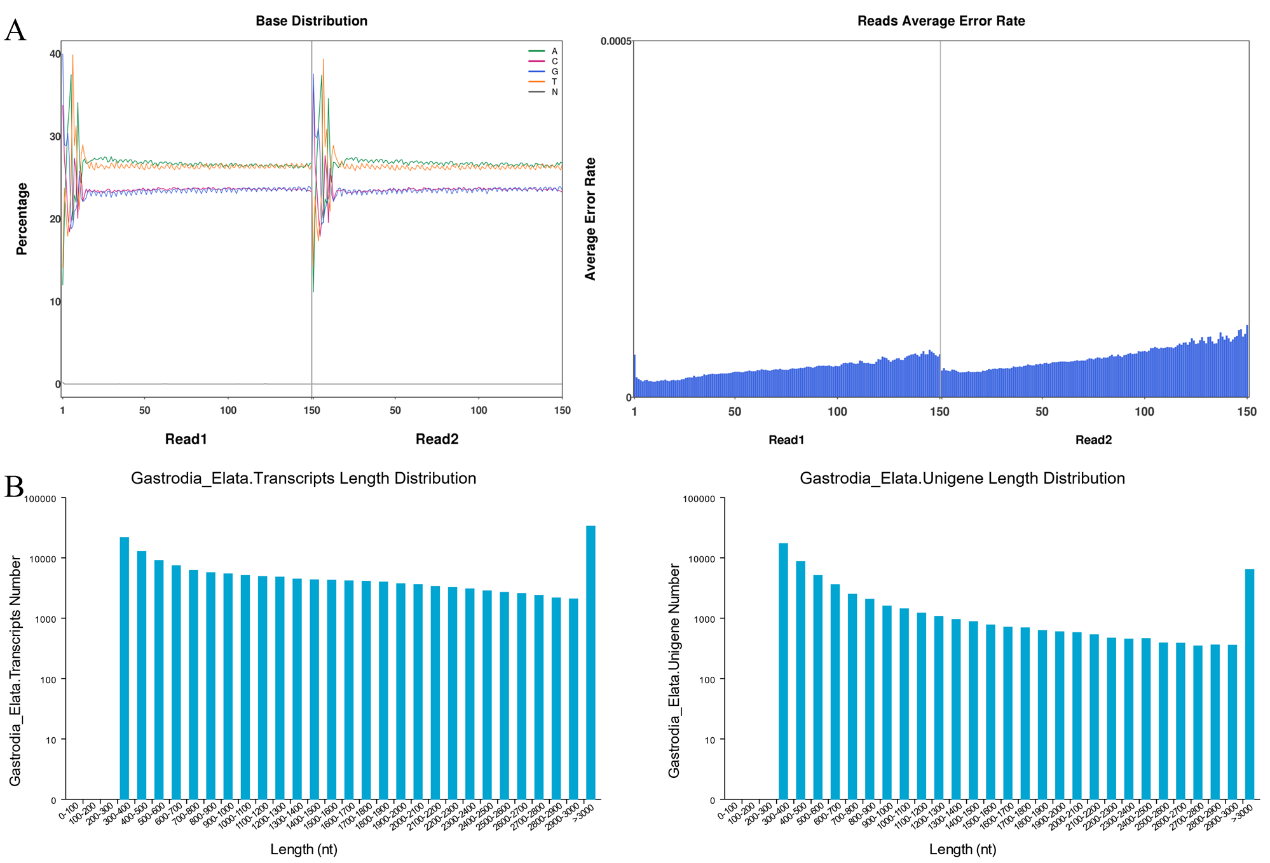


**Figure S1** (**a**) Base distribution and reads average rate of raw data. (b) Transcripts and Unigenes length distribution after *de novo* assembly.
