# Supplementary material for: Transcriptome analysis reveals underlying immune response mechanism of fungal (Penicillium oxalicum) disease in Gastrodia elata Bl. f. glauca S. chow (Orchidaceae)
Source: BMC Plant Biol. 2020 Sep 29;20:445. doi: 10.1186/s12870-020-02653-4 (PMC7525978; doi:10.1186/s12870-020-02653-4)
Supplement: Supplementary file 8 — Additional file 8: Figure S2. Monthly values accumulated from 1981 to 2010 in Jingyu County, Baishan City, Jilin Province, PR China. [file 12870_2020_2653_MOESM8_ESM.docx]

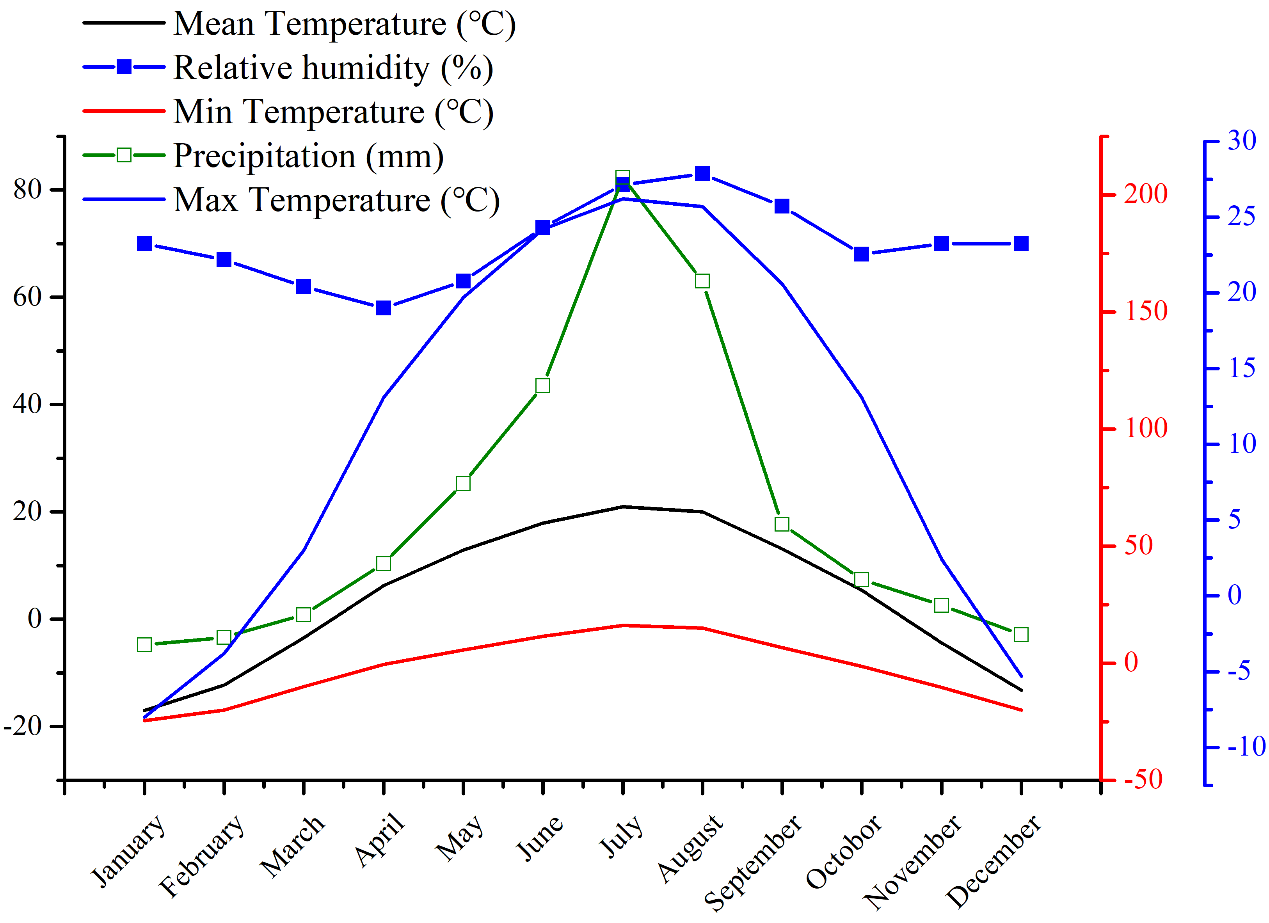


**Figure S2** Monthly values accumulated from 1981 to 2010 in Jingyu County, Baishan City, Jilin Province, PR China.
